# Using Eye Trackers for Usability Evaluation of Health Information Technology: A Systematic Literature Review

**DOI:** 10.2196/humanfactors.4062

**Published:** 2015-04-14

**Authors:** Onur Asan, Yushi Yang

**Affiliations:** ^1^ Center for Patient Care and Outcomes Research Department of Medicine Medical College of Wisconsin Milwaukee, WI United States; ^2^ Department of Industrial Engineering Clemson University Clemson, SC United States

**Keywords:** health information technology, eye-tracking technology, usability evaluation

## Abstract

**Background:**

Eye-tracking technology has been used to measure human cognitive processes and has the potential to improve the usability of health information technology (HIT). However, it is still unclear how the eye-tracking method can be integrated with other traditional usability methodologies to achieve its full potential.

**Objective:**

The objective of this study was to report on HIT evaluation studies that have used eye-tracker technology, and to envision the potential use of eye-tracking technology in future research.

**Methods:**

We used four reference databases to initially identify 5248 related papers, which resulted in only 9 articles that met our inclusion criteria.

**Results:**

Eye-tracking technology was useful in finding usability problems in many ways, but is still in its infancy for HIT usability evaluation. Limited types of HITs have been evaluated by eye trackers, and there has been a lack of evaluation research in natural settings.

**Conclusions:**

More research should be done in natural settings to discover the real contextual-based usability problems of clinical and mobile HITs using eye-tracking technology with more standardized methodologies and guidance.

## Introduction

Health information technology (HIT) systems are promising tools for improving quality, patient safety, and efficiency in health care systems [1-4]. This technology has been widely adopted due to governmental incentives, including funding, over the past few years [5]. However, despite powerful external forces driving the adoption of HIT, research has shown that physicians are still unsatisfied with, or resistant to, the technology [6] due to several unintended consequences from workflow and design-/usability-related problems. For example, one study reported that physicians felt that the standard reports produced by HIT systems actually reduced the usability and transparency of medical records [7]. To address usability issues and improve the design of HIT, usability evaluation research is necessary and becoming more prevalent [8,9]. Eye-tracking technology is one important tool that will be essential in such usability research.

Eye-tracking technology has been used to measure cognitive processes since the 1970s [10]. However, it has not been widely used for research purposes until recently, when the reduced cost of the equipment and user-friendly analysis tools made eye-tracking technology more readily available to researchers [11]. Eye-tracking technology is promising in HIT usability research because of the close relation between visual stimuli and attentional mechanisms. Based on human information processing theory, people can only attend to a certain amount of visual stimuli at a time, due to a limited amount of mental resources [12]. Excessive information stimuli will result in mental overload that is correlated to physiological changes, such as pupil diameter [13]. Therefore, by tracking infrared light that is reflected by the human eye, we can understand a participant’s mental load and cognitive state [14]. We can also detect the areas on a user interface that may capture users’ attention and are processed by the human brain [15].

Two important measurements of eye-tracking technology are fixation and saccade [16]. Fixation has been operationally defined by previous researchers as a gaze that is longer than 300 milliseconds [17]. Fixation describes the moments when a human’s eyes are relatively stationary, indicating the moments when the brain processes information received by the eyes [18]. Different patterns of fixation indicate different forms of human information processing. For example, high fixation rates usually indicate an area of great interest, which attracts the user’s attention [19], whereas extremely long fixations indicate uncertainty and difficulties with information processing [10]. In addition, successive fixations are indications of inefficient visual search [20]. Saccades happen between fixations, when rapid eye movements shift attention from one target to another [18]. Saccade initiates when a critical cognitive event occurs and represents an attention shift [21].

Eye-tracking data can be integrated, synthesized, and visualized using software suites, such as commercially available analysis tools. Different types of visualizations, such as heat maps and gaze plots, communicate different types of information [22]. A heat map shows the observed areas and unobserved areas on an interface in different colors [23]. A gaze plot displays gaze motions by representing the sequence of saccades and fixations in the form of a scan path [24]. These visualizations are useful for explaining the user experience and usability of user interface design, and they help us make decisions on how to optimize the elements on that interface [25,26]. For example, heat maps and gaze plots have been used to determine certain areas of a webpage that attract the attention of viewers [27]. They have also been used to evaluate the usability of cartographic animations on interactive maps [28,29].

Based on the International Organization for Standardization (ISO) Standard, usability is the extent to which users can achieve a goal effectively, efficiently, and with satisfaction [30]. Due to the fact that eye-tracking measurement is closely related to attentional mechanisms and is able to accurately reveal cognitive processes, eye-tracking technology could play a more important role in this essential procedure for evaluating HIT. Yet, thus far it has been used minimally in usability evaluation studies. The objective of this literature review is to report and understand the current state of HIT usability evaluation studies that have used eye-tracker technology, and to envision the potential use of eye-tracking technology in future research.

## Methods

### Selection Strategy

We conducted a systematic online database search to identify articles published before September 2014 that were relevant to the aims of this study. Articles were included as indexed in four reference databases: Medline, Web of Science, ScienceDirect, and PsycINFO. Broad keyword searches were used to identify relevant articles in each database. Each initial search focused on one of three key components: (1) a word or phrase related to usability evaluation, (2) a word or phrase related to HIT, or (3) a word or phrase related eye-tracker technology.

Keywords related to usability evaluation included usability testing, user experience, user test, user-centered design, system design, interface design, and interaction design. Keywords related to HIT included health IT, health informatics, health technology, medical technology, eHealth, telemedicine, communication tools, educational technology, decision support technology, health app, and wearable technology. Keywords related to eye-tracker technology included eye-tracking technology, eye tracker, Tobii, Sensomotoric Instruments, eye movements, gaze, eye fixation, and saccade. We also identified potentially eligible articles by manual literature searches, by examining article reference lists and by searching in Google Scholar.

### Inclusion and Exclusion Criteria

We initially defined the scope of the review by determining inclusion and exclusion criteria. Papers were included if they contained all of the following: (1) the research used eye-tracking technology as a data collection tool, (2) the research evaluated an HIT with users, and (3) the research explicitly mentioned the improvement of HIT usability based on the eye-tracking data. Papers were excluded if they (1) were not in English, (2) were published 10 or more years ago (ie, prior to 2004), (3) did not evaluate an HIT, (4) focused on technologies not related to health care, (5) used eye-tracker technology for some purpose other than data collection (ie, as an input device), or (6) did not mention any indications of the system usability based on the eye-tracker data.

### Analysis

Based on the methods-description approach, we analyzed the selected papers that met the inclusion criteria [31]. Key article characteristics were recorded using a template with the following sections: title, author, purpose, and key findings [31]. After the creation of the table, we captured key data by coding as the recurrent topics. Coding is an analytical process that allows the articles to be categorized based on factors that are thought to be important [32]. Through the coding process, the following topics were explored: the research question answered by eye-tracking data, types of health IT to be evaluated, evaluation apparatus, eye-tracker measurement and analysis, and how eye-tracker technology is combined with other usability evaluation methods.

## Results

### Overview

**Figure 1:**
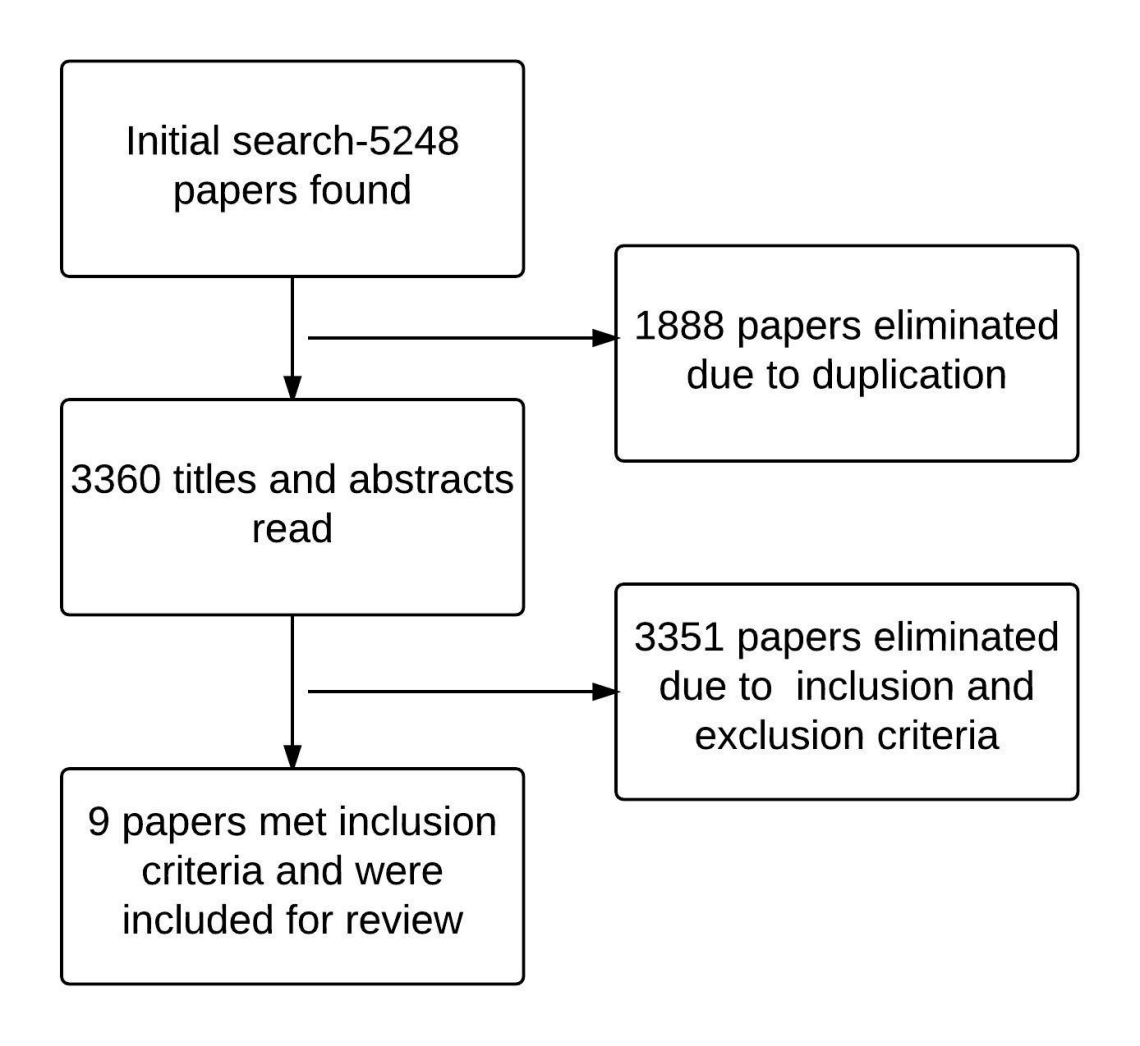
Flow diagram of the study selection process.

A total of 5248 papers were found by using the search terms and databases described above. Of these, 1888 papers were removed due to duplication. After reviewing the titles and abstracts based on inclusion and exclusion criteria, we eliminated another 3351 papers. This resulted in a total of 9 papers remaining for this review (see Figure 1). An overview of the 9 papers can be found in Table 1 [33-41]. It is important to note that 2 of the 9 papers are from the same project [35,36]. We included both because they fit the inclusion criteria. Of these, 1 paper describes one of the earliest studies using eye-tracking technology to evaluate the usability of a computer application [35], and the other is a complete report of the whole user-centered design process, which reflected more information on the entire research context [36].

All selected papers discussed user evaluation of a type of HIT using eye-tracking technology as a data collection tool. Of the 9 papers, 3 of them (33%) mainly discussed a usability evaluation of an HIT using eye-tracker technology [33,35,40]. Of the 9 papers, 3 of them (33%) presented an entire user-centered design process and discussed the usability evaluation of an HIT using eye-tracking technology as one part of the paper [34,36,41]. For instance, 1 study discussed how focus groups were used as a way to develop a quality-of-life support prototype, and then evaluated the usability of the prototype using eye-tracker technology [41]. The main purpose of the 3 remaining papers was not usability evaluation of the HIT, however, the eye-tracking data derived from the user evaluation clearly provided a basis for usability improvement [37-39]. For instance, 1 study reported that providers did not recognize patient-identity errors on a computerized provider order entry (CPOE), even if the eye-tracking data indicated that they looked at the area that contained errors [37]. These results have been translated to usability improvement recommendations for the system, for example, to make the important identity information more salient on the interface.

**Table 1:** Summaries of papers used in the review.

Author and reference	Title	Purpose	Key findings
Bansback et al [33]	Development and preliminary user testing of the DCIDA (Dynamic computer interactive decision application) for ‘nudging’ patients towards high quality decisions	To develop and test a computer application that enhances conventional patient decision aids so that common decision errors made by patients can be reduced.	The Dynamic Computer Interactive Decision Application (DCIDA) version of patient decision aids was understandable to users and it was able to help users focus on attributes that are of individual importance to them.
Barkana and Acik [34]	Improvement of design of a surgical interface using an eye tracking device.	To use eye-tracking technology to improve the design of a surgical interface to obtain the optimum configuration.	The interface of the early version of a surgical interface was redundant. With two larger scans at higher spatial resolution on the interface, participants were able to complete tasks more quickly, and the visual acquisition corresponded more to the natural visual search.
Eghdam et al [35]	Combining usability testing with eye-tracking technology: evaluation of a visualization support for antibiotic use in intensive care	To investigate if Infobiotika supports efficient and effective navigation and to observe the user's navigation paths, visual scan patterns, and distribution of visual attention.	Infobiotika was effective and efficient in terms of navigation support, and was a learnable product for intensive care unit (ICU) physicians.
Forsman et al [36]	Integrated information visualization to support decision making for use of antibiotics in intensive care: design and usability evaluation	To investigate the role of visualization as a method to support intensive care physicians’ decision making about antibiotic use, analyze users’ work processes and information needs, develop an interactive tool for integrated information visualization, and perform usability testing.	The visualization tool was usable for supporting ICU physicians in antibiotic use. Physicians had increased awareness of a patient's infection-related data and felt more in control of the situation.
Henneman et al [37]	Providers do not verify patient identity during computer order entry	To determine the frequency of verifying patient identity in an emergency department (ED) during computerized provider order entry (CPOE).	Medical providers did not usually verify patient identity prior to selecting the patient from the list and ordering tests. They often did not recognize patient-identity errors in the system.
Kules and Xie [38]	Older adults searching for health information in MedlinePlus – an exploratory study of faceted online search interfaces	To examine how searchers interact with a faceted Web-search interface.	Faceted interfaces played a substantial role in participants' use of the search result pages. The severity of the health condition affected the use of faceted interfaces.
Liu et al [39]	The use of illustration to improve older adults’ comprehension of health-related information: Is it helpful?	To examine whether explanatory illustrations can improve older adults' comprehension of written health information.	Older adults had difficulties understanding the illustrations as well as integrating the illustrations with the text. Older adults did not benefit from the use of illustration.
Rashid et al [40]	Preliminary usability testing with eye tracking and FCAT analysis on occupational safety and health websites	To measure effectiveness, efficiency, and satisfaction of the Occupational Safety and Health (OSH) website, and to gather user feedback.	Eye-tracker data and user feedback helped identify usability problems of three OSH websites.
Wolpin et al [41]	Development and usability testing of a web-based cancer symptom and quality-of-life support intervention	To develop a user-centered prototype, and assess user preferences from usability testing of a revised prototype of the Electronic Self-Report Assessment for Cancer-II (ESRAC 2.0) project.	An application was developed that integrated the patients’ needs through the methods of participatory design, usability testing, and iterative development.

### What Research Questions Are Answered by Eye-Tracker Technology?

We identified different research questions that are answered by eye-tracker technology in the selected papers. The first question that can be answered by eye-tracker technology is whether the user experience and performance using an HIT has been improved based on the eye-gaze patterns, which primarily reflects the system effectiveness, efficiency, and user satisfaction [33-36,40,41]. The second question that can be answered by eye-tracker technology is how people use visual cues in the decision-making process, which primarily reflects the linkage between human visual stimulus and cognitive processing [37]. The third question that can be answered by eye-tracker technology is how information is processed differently under different circumstances, such as age and health conditions, which primarily reflects the variability of human performance [38,39].

### What Types of Health Information Technologies Were Evaluated Using an Eye Tracker?

We identified different types of HITs in the selected papers. In terms of functionality, the technologies included online health information website interfaces [38-40], surgical interfaces [34], decision support systems [33,35,36], computerized provider order entry systems [37], and symptom and quality-of-life information systems [41]. In terms of target users, the health care information technologies were for the general public [38-40], patients [33,41], and physicians [34-37].

### What Is the Experimental Apparatus of the Usability Test?

We identified different experimental apparatuses of the user tests. Researchers evaluated HITs in the forms of developed computer website/application [33,34,38,40], simulated prototype [35,36,41], and screenshots [37,39]. Researchers used three different kinds of eye trackers to collect data: on-screen eye trackers (Tobii T60 and T120) [33,38,40,41], mobile eye trackers that are external to a personal computer (Sensomotoric Instruments [SMI] 500, Tobii X-60 and X120) [34-36], and head-mounted eye trackers [37,39]. Experiments were conducted either in a usability lab room or a meeting room. None of the experiments were conducted in the natural setting.

Out of the 9 papers, 2 of them (22%) reported a failure to collect eye-tracking data during the usability test [37,39]. Of those 2 papers, 1 of them reported that data for 12 out of 250 patient identification scenarios were not recorded due to failures in the eye-tracking system [37]. The other paper reported that the eye tracker was not able to perform for one-third of the older adult participants [39]. Both papers used a head-mounted eye tracker for data collection.

### What Did the Eye Tracker Measure and How Was Data Analyzed?

We identified three basic eye-tracker measurements in our selected papers. The measurements included fixation duration [33,34,38-40], the locations of eye movement [35-37], and the fixation count in an area of interest [34,38]. Some papers included two measurements, focusing on both fixation duration and number of fixations in an area of interest [34,38]. Three basic methods were also used to analyze the eye-tracker measures in the selected papers, including heat map [33,34,40], gaze plot [35,36,41], and statistical analysis [34,37-39]. Generally, a heat map is used when fixation-duration data is collected [33,34,38], a gaze plot is used when the location of eye-movement data is collected [35,36], and statistical analysis is used when fixation-duration data is collected [34,38,39]. The heat map and gaze plot are qualitative methods for understanding the observed areas and gaze motions on an interface. Statistical analysis is a quantitative method to examine the effects of two different versions of a design or two different user groups on the task completion time.

### How Is Eye-Tracker Technology Combined With Other Usability Methods?

The selected papers also showed other usability evaluation methods that are combined with eye-tracker data to explore usability problems in HIT systems. The methods include the System Usability Scale (SUS) [33,35,36], the think-aloud protocol [33,38,41], the National Aeronautics and Space Administration Task Load Index (NASA-TLX) and Short Post-Assessment Situational Awareness (SPASA) questionnaire [34], posttest interviews [36], metrics measurement [39], and Feedback Capture After Task (FCAT) [39]. There are two different types of think-aloud evaluations: concurrent think aloud, which encourages participants to tell what they think while using the program, and retrospective think aloud (RTA), which asks participants to verbalize their thoughts afterwards. Researchers in selected papers used concurrent think aloud [41], RTA [38], and a combination of both [33]. Table 2 shows the research questions that were answered by eye-tracker technology in the selected papers.

**Table 2:** Summary of research questions.

Questions and answers			Reference
**Q1: What research questions are answered by eye-tracker technology?**			
		System effectiveness, efficiency, and user satisfaction	[33-36,40,41]
		Linkage between human visual stimulus and cognitive processing	[37]
		The variability of human performance	[38,39]
**Q2: What types of HITs were evaluated using an eye tracker?**			
	**Technology type by functionality**		
		Health information website interfaces	[38-40]
		Surgical interfaces	[34]
		Decision support systems	[33,35,36]
		Computerized provider order entry systems	[37]
		Symptom and quality-of-life information systems	[41]
	**Technology type by target users**		
		General public	[38-40]
		Patients	[33,41]
		Physicians	[34-37]
**Q3: What is the experimental apparatus of the usability test?**			
	**Experimental apparatus by technology**		
		Developed computer program	[33,34,38,40]
		Simulated prototype	[35,36,41]
		Screenshots	[37,39]
	**Experimental apparatus by eye tracker**		
		On-screen eye tracker	[33,38,40,41]
		Mobile eye tracker	[34-36]
		Head-mounted eye tracker	[37,39]
**Q4: What did the eye tracker measure and how was data analyzed?**			
	**Eye-tracker data collected**		
		Fixation duration	[33,34,38-40]
		Eye movement location	[35-37]
		Fixation count in area of interest	[34,38]
	**Eye-tracker data analyzed**		
		Heat map	[33,34,40]
		Gaze plot	[35,36,41]
		Statistical analysis	[34,37-39]
**Q5: How is eye-tracker technology combined with other usability methods?**			
		Think-aloud protocol	[33,38,41]
		System Usability Scale	[33,35,36]
		Questionnaire	[34]
		Posttest interview	[36]
		Metrics measurement	[39]
		Feedback Capture After Task	[39]

## Discussion

### Principal Findings

The purpose of this literature review was to examine usability evaluations of any type of HIT using eye-tracking technology. This review also aimed to identify the research gap and potential uses of eye-tracker technology in future HIT research. This review was conducted based on the inclusion and exclusion criteria specified in the Methods section. Based on the results, we determined that, although eye trackers provide rich data for the improvement of HIT systems, the use of eye trackers for usability evaluation of HITs is still in its infancy, as only 9 papers were found that fit within the inclusion criteria.

We organized the results into five main questions: (1) What research questions are answered by eye-tracking technology?, (2) What types of health care information technologies were evaluated using an eye tracker?, (3) What was the experimental apparatus of usability evaluation?, (4) What did the eye trackers measure and how was data analyzed?, and (5) How was eye-tracker technology combined with other usability methods?

Papers that were included in this review had different purposes and research goals. The types of HITs evaluated were limited, resonating with our finding that the use of eye trackers for the evaluation of health IT is in an early stage. However, eye trackers are becoming a promising tool for usability studies, as demonstrated by the increasing number of research studies in recent years. We also found that researchers used various means of data collection and analysis using eye trackers. On the one hand, this demonstrates the rich variety of data that can be captured by eye trackers and the flexibility of interpretation of eye-tracker data. On the other hand, it shows the lack of a consensus on how to conduct user evaluation of HITs using eye trackers at this stage. In addition, we found that eye-tracking technology, as a part of usability evaluation methodology, was supplemented by other traditional methods. Generally, eye-tracking data can reveal the patterns of user difficulties when completing tasks using HIT, while other supplemental inquiries are used to unfold the reasons behind those patterns. Therefore, eye-tracking technology has to integrate with other techniques, as most physiology measurements do, because eye-tracking technology alone cannot tell the entire story.

### Different Research Questions

The reviewed papers reflected different research questions that were answered by eye-tracking technology. Of the 9 papers, 6 of them (67%) were directly related to the system usability, focusing on the efficiency, effectiveness, and satisfaction when completing tasks with a specific HIT. Of these, 1 paper was related to the examination of a gap between visual and cognitive process. For example, a user missed information because he/she did not pay attention to it, even if eye-tracking technology suggested that the user had seen that information [37]. Another 2 papers (22%) out of 9 were related to the evaluation of different gaze patterns under different circumstances. For example, age had been identified as a factor for processing information [39]. Although the research questions were different, all of these studies commented on how eye-tracking data might have direct or indirect implications for the usability improvement of the evaluated HIT.

### Limited Types of Health Information Technologies

The reviewed papers involved five different types of HITs, including 3 out of 9 papers (33%) evaluating health information website interfaces, 3 papers (33%) evaluating decision support systems, 1 paper (11%) evaluating a surgical interface for physicians, 1 paper (11%) evaluating a computerized provider order entry system for physicians, and 1 paper (11%) evaluating a symptom and quality-of-life information system for physicians. The reviewed papers involved three different types of users, including 3 papers (33%) for general public health IT, 2 papers (22%) for patients, and 4 papers (44%) for physicians. Thus far, eye trackers have been used most often to evaluate health information website interfaces. This indicates that evaluating a website interface using eye-tracker analysis may provide rich theoretical guidance and reveal available practices that researchers can refer to [42,43]. Moreover, the methods for evaluating a website interface are familiar to usability specialists.

However, there is much potential for eye-tracker technology to be applied to other types of health IT as well. One particular aspect of health IT that lacks usability research using eye-tracker technology is electronic health record (EHR) systems. EHR systems have helped to revitalize physician and nursing practice, and have the potential for positive impact on clinical processes in terms of efficiency, productivity, and patient safety [44]. Health care providers’ attitudes toward EHR systems have been assessed and results showed that a majority recognized the positive influence of EHR systems in terms of decreased workload, improved quality of documentation, and electronic charting [45]. However, some other studies also reported a negative impact of EHRs, such as workflow interruptions and introduction of new errors because of usability factors, which have also been identified as a major barrier for successful EHR implementation [46,47]. Eye-tracking technology can also be used to identify usability problems to improve the design in a better way.

Another gap exists in the application of eye-tracking technology to usability studies of novel consumer HITs. Health apps and devices are becoming prevalent in the market. Devices such as the iPad, iPhone, iPod Touch, and Apple Watch have been the target devices to provide a richer and more convenient user experience of health care information technology [48]. Wearable interfaces and Web-based activity-monitoring systems are popular in the current market for the encouragement, persuasion, and guidance of healthy lifestyles. Because of the smaller size of these screens, there are increased difficulties for users to operate these systems and for designers to maximize the available screen area effectively [49]. Also, users expect to interact with these HITs in ways that are consistent with other technologies, without the need to read instructions. Eye-tracking technology has the ability to examine whether this has been achieved [50]. In that regard, eye-tracking data would be very helpful in understanding how users interact with those technologies and in providing designers with the basis to make improvements.

### Lack of Research in a Natural Setting

In terms of the prototypes that were evaluated in the reviewed papers, a majority of them (4/9, 44%) evaluated developed computer programs that have already been adopted in health care systems. Of these papers, 2 of them evaluated screenshots of the real websites, and 2 of them evaluated usability using a simulated prototype in high fidelity. Unlike many other usability techniques, such as the formative usability evaluation approach, that are primarily introduced in the early phase of the user-centered design process, we found almost all the papers evaluated HITs at a very late phase or even after implementation, as a summative approach. The benefit of doing a summative usability evaluation is that researchers are able to create an approximation of the actual use scenario of HITs. Compared with low-fidelity, nonfunctional prototypes, such late-phase testing is more likely to uncover real usability problems [51]. However, even such high-fidelity approximations fall short of researching HIT use in the natural setting. The health care system is a sociotechnical system with a complex structure, complex dynamics, and multiple stakeholders [52]. Not until health care providers work in the real environment can many organizational issues emerge, such as patient privacy, workflow complexity, and disruptions [52-57]. Those factors influence the usability of HIT in ways that cannot be captured by lab-based evaluations. Therefore, an ecological gap is a particular concern for HIT evaluation representing the differences of user study results between the lab and the real setting [58]. Because of this, we believe there are certain usability problems of HIT that can only be discovered in the field within the real context of HIT use.

Unfortunately, at this point there has been no usability evaluation conducted using eye-tracking technology in real settings. All of the reviewed papers conducted user studies in a meeting room or a usability lab. Possible reasons for this gap could be the mobility limitations of eye-tracker technology, the possible intrusion of such technology on work, technical difficulties, and the calibration process of the eye-tracking equipment. Nearly half of the papers (4/9, 44%) used eye trackers that were embedded within a computer screen, which are impossible to move into real settings. Of the 9 papers, 2 of them (22%) used head-mounted eye trackers, which are easy to move but intrusive to the health care provider’s work if the evaluation is conducted in the field, negatively influencing the work in that time-sensitive environment. Moreover, such head-mounted trackers are more likely to have technological difficulties, which risk accurate data collection. Of the 9 papers, 3 of them (33%) used mobile eye trackers, which are probably the best equipment to be incorporated into field research in the real HIT setting. However, the calibration process may add additional steps to the already complex workload of nurses or physicians. Moreover, it is unlikely that a health care provider will stay in one place for a long period of time, and their movements will disrupt the calibration [59]. Despite this, we still believe in the necessity and value of conducting real-life usability evaluations of health IT using eye trackers. We expect advancements in eye-tracking technology to address this obstacle. For example, a new technology—Glasses—is capable of collecting data in real settings without the problems of calibration or too much intrusion on the health care provider’s work.

### Gaps of Eye-Tracker Data Analysis

We found that the eye-tracker measurements in the reviewed papers were mainly fixation and saccade, which supports the finding by Poole and Ball [18]. More than half of the reviewed papers (5/9, 56%) collected fixation-duration data. For example, researchers used fixation duration as an indicator of the efficiency of human interaction with the surgical interface [34]. Of the 9 papers, 3 of them (33%) collected eye-movement locations. Of these, 2 papers collected both the fixation duration and fixation count in areas of interest. For example, researchers evaluated a Dynamic Computer Interactive Decision Application, using fixation number and fixation duration as indicators of attributes on the DCIDA [33]. We found that certain quantitative eye-tracker data are more favored by researchers, such as fixation duration and fixation count. Qualitative data collection and analysis appeared less frequently in the reviewed papers, which corresponds to the finding by Yen and Bakken [9].

Qualitative analysis is becoming prevalent partly because of the improvement of software suites, making the analysis easier and less intensive. Of the 9 papers, 3 of them (33%) translated the data into qualitative visualization, such as heat maps and gaze plots. While statistical analysis is powerful in comparing completion time and errors, it is only part of the usability evaluation. For a full usability evaluation, we believe the qualitative data in visualization can illustrate more usability problems. Using a heat map, it is easy to determine if specific content is usable or not. Using a gaze plot, it is possible to determine if users follow an efficient and predetermined route when searching for specific information on the interface.

However, we found that the interpretation of such visualizations lacked scientific guidance based on an established theoretical method, so interpretations tended to seem arbitrary and subjective. At this point, researchers are struggling to find a theory or a commonly used procedure to guide the interpretation of heat maps and gaze plots. Therefore, we expect that in the future a more structured system of interpretation will be developed for heat maps and gaze plots.

### Opportunities for Integration

With the visualization of eye-tracker data, researchers can identify the areas of an interface that have created difficulties in participants’ minds. However, based solely on the eye-tracker data, there is no way to understand the precise cognitive reasons behind a participant’s eye-gaze patterns. For example, there might be many possibilities for an eye fixation, such as fatigue, distraction, confusion, and engagement [18,60]. Therefore, researchers will have to integrate other quantitative and qualitative research methods with eye-tracking research in order to understand why people behave in a particular way. Of the 9 papers, 7 of them (78%) used other methods along with the eye tracker, some using more than one method. Of these, 3 papers used the think-aloud method, 3 papers used the SUS, 1 paper used the NASA-TLX and the SPASA questionnaire, 1 paper used a posttest interview, 1 paper used metrics measurements, and 1 paper used FCAT.

It is interesting that there are three different think-aloud methods used in the reviewed papers: concurrent think aloud, RTA, and a hybrid of both. The concurrent think-aloud method is the traditional method widely accepted and applied by usability evaluation researchers. It is a method that asks participants to verbalize their thoughts while interacting with the system [61]. However, the method has received criticism because the verbal process requires attention and may distract the participants [62]. Additionally, during the think-aloud method, users usually have the temptation to look at the researcher for conversation, which has the risk of disrupting the calibration of eye-tracking technology, thus causing researchers to lose eye-tracking data [63]. RTA records participants’ eye movements during the usability test session and then asks them to verbalize their thoughts afterward while watching the gaze-plot animation [64]. Research has shown that RTA enhances the validity and reliability of the usability evaluation results [65]. However, RTA does have some identified limitations, due to the limited capability of eye-tracker technology, which we need to be aware of. For example, eye trackers are not able to capture peripheral vision data. Although our peripheral vision is in low resolution, that still accounts for part of our visual input [11]. Similarly, orphan fixation can happen when the user is making some unintentional fixation or when the user looks at an area, but attention is somewhere else [59]. When researchers present this to the participant, it can surprise the participants and, therefore, distract them from an efficient RTA process [59].

Another reviewed paper used a hybrid of concurrent think-aloud and RTA methods [33]. The researchers asked the participants to think aloud while completing the task. However, if they could not think aloud about a particular page within 10 seconds, they would be asked to reflect after the task session. This method is superior because the participants have the opportunity to verbalize immediate thoughts during the evaluation session, but also have the opportunity to review and think more deeply after the test.

### Conclusions

Although eye tracking is a promising technology, the application of eye-tracking technology to health IT usability evaluation is still in its infancy, with limited theoretical guidance and practice. Therefore, we reviewed papers that were related to usability evaluations of HIT using an eye tracker, to understand the current state, identify the gaps, and envision future research. There is no doubt that eye-tracker technology would be able to provide valuable data if well-integrated with other traditional usability evaluation methodologies. However, the lack of field research of clinical and mobile HITs in natural settings is a huge gap that needs to be filled. Scientific guidance is also needed for the interpretation of eye-tracking visualizations. Eye trackers can play a significant role in the future of usability evaluations of HIT if they are used effectively and correctly.

## Abbreviations

CPOE: computerized provider order entry
DCIDA: Dynamic Computer Interactive Decision Application
ED: emergency department
EHR: electronic health record
ESRAC 2.0: Electronic Self-Report Assessment for Cancer-II
FCAT: Feedback Capture After Task
HIT: health information technology
ICU: intensive care unit
ISO: International Organization for Standardization
NASA-TLX: National Aeronautics and Space Administration Task Load Index
OSH: Occupational Safety and Health
RTA: retrospective think aloud
SMI: Sensomotoric Instruments
SPASA: Short Post-Assessment Situational Awareness
SUS: System Usability Scale
